# Checklists in Femur Fractures: High Adherence After Implementation of Computer-based Pediatric Femur Guidelines

**DOI:** 10.5435/JAAOSGlobal-D-21-00154

**Published:** 2021-08-09

**Authors:** Kyle Geiger, Oliver Orr, Andrew Gupta, Viviana Bompadre, Michael Goldberg, Ted Sousa

**Affiliations:** From the Washington State University (Dr. Geiger), Elson S. Floyd College of Medicine; the Seattle Children's Hospital (Dr. Orr, Dr. Gupta, Dr. Bompadre, Dr. Goldberg, and Dr. Sousa); and the Shriners Hospital for Children (Dr. Sousa), Spokane, WA.

## Abstract

The American Academy of Orthopaedic Surgeons (AAOS) created an evidence-based clinical practice guideline for the care of pediatric diaphyseal femur fractures in 2010. Our institution implemented checklists based off these guidelines embedded in a standardized EMR order. The purpose of this study was to describe compliance with checklist completion and to assess safety improvement in a large urban pediatric hospital.

**Methods:**

Retrospective and prospective data were collected from 2 years before and 5 years after checklist implementation. This included the patient safety checklists from August 2011 through August 2016. Patients aged 0 to 18 years with a diaphyseal femur fracture were queried from the EMR and included in this study. Patient charts were reviewed for complications, including nerve injury, pressure sore, leg length discrepancy, loss of reduction, failure of fixation, nonunion, delayed union, and infection. Compliance rates were reported based on the AAOS clinical practice guidelines.

**Results:**

A total of 313 patients for the postchecklist period were reviewed in this study. Of 219 patients eligible for inclusion, 198 had checklists completed (group B). This group was compared with 100 patients with diaphyseal femur fractures from the period before implementation of the checklist (group A). We found no statistical difference in the number of patients with complications between groups (12% in both groups, *P* = 0.988). Postoperative checklists demonstrated that 89.9% of patients (178/198) received age-appropriate treatment consistent with the AAOS guideline recommendations after implementation of the checklist. Before the checklist implementation (group A), 94% (94/100) adhered to the guidelines.

**Conclusion:**

This study reveals high compliance rates with the AAOS evidence-based clinical practice guideline for the management of pediatric femur fractures. Implementing standardized checklists is possible by embedding them into the EMR. Implementation of checklists did not improve compliance or patient outcomes.

From 1990 through 1996, Hinton et al^1^ recorded the annual rate of children with femoral shaft fracture to be 19.15 per 100,000. Pediatric femoral shaft fractures are uncommon but have the potential to require surgery with long-term complications.^2^ This presents an opportunity where optimizing clinical decision making based on current evidence is crucial to patient safety. In 2010, the American Academy of Orthopaedic Surgeons (AAOS) and the Pediatric Orthopaedic Society of North America released evidence-based clinical practice guidelines (CPG) for the management of pediatric diaphyseal femur fractures.^3^ These guidelines outline an age-based approach to pediatric diaphyseal femur fractures, giving suggestions with varying strength of recommendation.

There is minimal information in the literature on adherence to AAOS guidelines and checklists. Although care has been shown to be consistent in the youngest age groups, there is a large amount of variability among institutions.^4^ In general, CPG are intended to reduce clinical variability; however, one recent study found little direct clinical effect of AAOS CPG on the treatment of pediatric diaphyseal femoral fractures.^5^

In 2011, our institution implemented an EMR-based patient safety checklist in an effort to improve compliance with the AAOS guidelines. The purpose of this study was to describe compliance with checklist completion and assess safety improvement in a large urban pediatric hospital. Our hypothesis was that the implementation of a computer-based checklist for pediatric femur fractures does not improve compliance with AAOS guidelines or safety in a large urban pediatric hospital.

## Methods

In 2011, our institution created a fracture safety checklist for pediatric femur fractures modeled after the AAOS guidelines. After IRB approval, patients aged 0 to 18 years with a confirmed diaphyseal femur fracture from August 2009 through August 2016 were queried by ICD-9 and ICD-10 codes from the EMR and included in this study. Patients were excluded if they had a diagnosis of osteogenesis imperfecta (OI), skeletal dysplasia, or pathologic fracture. Retrospective and prospective data were collected from 2 years before (2009 to 2011) and 5 years after (2011 to 2016) the checklist implementation. The retrospective data were limited to 2 consecutive years earlier because of a change in EMR and happened to be exactly 100 patients. Patients aged 0 to 18 years with a diaphyseal femur fracture were queried from the EMR and included in this study. The written explanations for deviating from standard of care when the guidelines were not followed were gathered and evaluated. In addition, patients were excluded if the fracture was not diaphyseal or initially presented/treated to an outside hospital. Compliance rates were reported based on adherence to the AAOS clinical practice guideline. Complications reviewed were infection, pressure sore from cast, nerve injury, failure of fixation, anesthetic complication, medical complication, delayed union, nonunion, leg length discrepancy (defined as >2 cm length discrepancy), and loss of reduction from spica cast. Patient charts were reviewed for return to the operating room with reason also collected. Return to operating room included unplanned return to operating room and did not include planned hardware removal. To assess safety, we collected data from eligible patients 2 years before the implementation of the checklist and compared both groups.

The checklist seems as a hard stop in the EMR that forces the provider (in most cases, the PGY-3 resident physician) to fill out the form to continue with patient care. The trainee was unable to put in order on an order set until the questions had been answered. Providers were instructed on the use of the checklist at the time of implementation. The checklist includes assessment for radiographic evaluation of fracture, possible pathologic fracture, evidence for nonaccidental trauma (NAT), associated head injury, and age-appropriate treatment. If AAOS guidelines are not followed and deviates from the checklist, the resident must provide a written explanation for deviating from standard of care. After completion, the attending physician receives a notification to attest the completed checklist.

Baseline demographic characteristics for prechecklist and postchecklist groups were assessed using the Student *t*-test for continuous variables and the chi-square test for categorical variables. Statistical significance was set at *P* < 0.05 and all confidence intervals at 95%.

## Results

A total of 313 patients for the postchecklist period were reviewed in this study. Patients were excluded if they had a diagnosis of OI, skeletal dysplasia, or pathologic fracture. Of those 219 patients eligible for inclusion, 198 had checklists completed (group B). To assess safety, this group was compared with 100 consecutive patients with diaphyseal femur fractures from the period before implementation of the checklist (group A). An attempt was made to find more prechecklist patients; however, this was made difficult by the lack of complete medical records. Patients in both groups did not differ in age (4.55, range 0 to 16 in group A; 4.48, range 0 to 17 in group B, *P* = 0.836), sex (81% male patients in group A and 77% in group B, *P* = 0.364), or follow-up (276 ± 7 days in group A and 222 ± 4.7 days in group B, *P* = 0.465) (Table 1).

**Table 1:** Demographics and Complication Rates Between Groups

Factor	Group A (Prechecklist) (n = 100)	Group B (Postchecklist) (n = 198)	*P* value
Age	4.55 (0-16)	4.48 (0-7)	0.836
Sex (M)	81 (81%)	152 (77%)	0.364
Follow-up (days)	276 ± 7	222 ± 4.7	0.465
No. of patients with complications	12 (12%)	23 (12%)	0.988
No. of patients who return to OR	2 (2%)	5 (3%)	0.608

Checklists were offered and completed at multiple time points, in the ED 24% (48/198), preoperatively in 95% (189/198), and postoperatively in 90% (178/198) of cases. Of patients eligible for the assessment of child abuse and head injury, 97% were evaluated appropriately (Table 2).

**Table 2:** Checklist Compliance

>ED Checklist (n = 48)	
AP lateral view	12 (25%)
Head injury assessment	46 (96%)
Child abuse	48 (100%)
Preoperative checklist (n = 188)	
Head injury assessment	183 (97%)
Child abuse	183 (97%)
Postoperative checklist (n = 177)	
Follow guideline treatment	112 (63%)

We found no statistical difference in the number of patients with complications between groups (12% in group A versus 12% in group B, *P* = 0.988). The return to OR rate was similar to both groups (2% for group A and 5% for group B, *P* = 0.608). Table 3 describes the type of complications found in each group. The “other” complications in the prechecklist group (1) included skin irritation from the Pavlik harness. “Other” complications in the postchecklist group (5 total) included hardware prominence requiring hardware removal (3), infection (1), and prolonged pain (1).

**Table 3:** Types of Complications by Group

Factor	Group A (prechecklist) (n = 12)	Group B (postchecklist) (n = 23)
Infections	1	0
Pressure sore	4	4
Nerve injury	0	0
Failure of fixation	1	2
Anesthetic	0	1
Medical	0	1
Delayed union	0	1
Nonunion	0	0
Leg length discrepancy (>1 cm)	1	6
Loss of reduction	3	3
Other	1	5

Regarding compliance, postoperative checklists demonstrated that 90% of patients (178/198) received age-appropriate treatment consistent with the AAOS guideline recommendations after implementation of the checklist. Twenty eligible patients did not have any checklist filled out during the study period. These patients were not included in the analysis for safety; however, we looked at the treatment provided to them and 6 of 20 differed from the guidelines. These 6 were stable fractures in patients under 2 years and amendable to discharge from the ED with minimal stabilization; hence, no checklist was filled out. The remaining (14/20) had followed the AAOS treatment guidelines; however, their checklist was bypassed because they went through their hospital course. We are unsure how the checklist was bypassed by the resident provider. We examined the treatment provided to the group of patients before the checklist implementation (group A), and we found that 94% (94/100) adhered to the guidelines. It is unlikely that more eligible patients were left out. Fractures were searched by CPT, ICD-9, and ICD-10 codes.

## Discussion

Checklists have been established as a way to potentially improve patient safety.^6-8^ The World Health Organization Surgical Safety Checklist is an example. The implementation of this checklist was associated with a reduction in the rate of death and inpatient complications.^9^ Other studies report notable barriers to checklist implementation, including duplication with existing lists, ambiguity of lists, and poor interprofessional communication.^10^ The creation of checklists generating “click fatigue” is also an important consideration.

Our study demonstrates that checklists can be easily followed and applied in large urban institutions that treat pediatric fractures through the use of a form embedded in the EMR. The success of these implemented checklists was also demonstrated by a recent study of the use of checklists for supracondylar humerus fractures.^11^ Similar to our report, Williams et al. concluded that their patient safety checklist did not affect patient care.

Of the patients for which surgery was indicated, postoperative checklists demonstrated that 90% of patients (178/198) received age-appropriate treatment consistent with the AAOS guideline recommendations.

This success is in contrast to the recently published literature by Oetgen et al^5^ at the Children's National Medical Center who report adherence varied from 22% to 94% depending on the recommended intervention. A much larger study published in 2019 by Roaten et al^12^ found notable deviation from the age-based treatment protocol. This multicenter study included 2,646 fractures and covered a longer period before and after the AAOS guideline publication (2004 to 2013). Roaten et al^12^ concluded that there were considerable variations in treatment methods and adherence to the guidelines and that there is a need for further studies to define optimal treatment. For example, they observed a notable increase in locked intramedullary nails in patients younger than 11 years. This is contradictory to the current AAOS CPG, but we believe it could be related to rising rates of childhood obesity in some geographical areas requiring more ridged nails. It is important to note that the study by Roaten did not look at safety and the differences in compliance may be related to the “culture” at each hospital.

Two main factors in the high adherence were likely mandatory completion of the checklist within the EMR and repeated education to increase awareness. Mandatory hard stops within the EMR at multiple points during admission ensure that the items on the checklist are not missed. Second, the ease of access to the checklist and guidelines plays a major role. A copy exists in the institution intranet and within the EMR. Continued education and integration within the EMR increase the checklist efficacy. Williams et al^11^ also discussed improvements to compliance such as more stringent requirements about the timing of checklist attestation and disabling the ability to simply close a checklist window before completion. Another major factor in checklist adherence was likely high compliance with guidelines before implementation (94%). We presume this is due to cultural, organizational, and financial effects, which may be difficult to quantify and measure. We also saw a drop in compliance from 94% to 90% and assume this to be because of a larger sample size and therefore more accurate measurement in the postchecklist group. Because of high compliance before the checklist implementation, we cannot conclude that checklists improve compliance.

The second goal of this study was to evaluate if these pathways improve patient care. This was done by assessing the femur fracture complication rate before and after the implementation of the patient safety checklist. No notable difference was observed in patient safety, as demonstrated by complications after implementation of the checklist. In future, given resources necessary to implement checklists, we recommend a thorough assessment of compliance rates to a problem to ensure a problem exists before implementing a checklist. Guideline adherence for NAT and head injury was also assessed. In the postguideline era, (NAT) and head injury was done in 97% of cases (178/183) compared with 48% reported in the study by Oetgen et al.^5^

There were several limiting factors in this study. This was a single-center study in a tertiary care pediatric hospital with a moderate number of cases reviewed. There were notable limitations to the assessment of complications because of low numbers. In addition, children with OI or cerebral palsy were excluded because the guidelines do not apply to them. However, a modified checklist could be developed as an extra safety measure to ensure they are evaluated for NAT, have proper imaging, and have treatment. An adapted checklist for these patients could be implanted into the existing pathway within the EMR. This also raises the question of whether customized checklists for providers based on their specific patient population would increase compliance with guidelines, as opposed to standardization.

A notable amount of time and effort was required to create checklists because EMR order sets had to be developed and implemented. Unfortunately, the time and resources used were not recorded. The authors persuade the residents to comply by asking them to fill out the checklist. New residents rotated into the hospital every 6 months. When the residents rotated into the hospital, an introduction to the checklist was made by using the PowerPoint. Why it was important to fill out the checklist was explained during the introduction. Because the checklist was part of an order set pathway, it was necessary to fill out the checklist to get the orders done at the same time. By this way, it was possible to ensure compliance and sustainability. Because of the notable effort and lack of improvement in patient safety/compliance, the checklist is no longer being used at our institution.

This study reveals high compliance rates with the AAOS evidence-based clinical practice guideline for the management of pediatric femur fractures. Implementing standardized checklists is possible by embedding them into the EMR. Implementation of checklists did not improve compliance or patient outcomes.
